# The establishment and functioning of the PRIMORE (European PRImary care MultiprOfessional REsearcher network) Project

**DOI:** 10.1017/S1463423619000276

**Published:** 2019-07-16

**Authors:** Frode F. Jacobsen, Mehmet Akman, Diederik Aarendonk

**Affiliations:** 1 Centre for Care Research – Western Norway, Western Norway University of Applied Sciences and VID Specialized University, Bergen, Norway; 2 Department of Family Medicine, Marmara University School of Medicine, Istanbul, Turkey; 3 European Forum for Primary Care, Utretch, The Netherlands

**Keywords:** primary health care, research, policy

## Abstract

There is a need for multiprofessional comprehensive studies to better understand the relationship between design and provision of primary care and long-term care and health outcomes. The PRIMORE (PRImary care MultiprOfessional REsearcher network) project aims at bringing together researchers with different backgrounds and from a wide range of professional groups within the fields of primary care research and long-term care research to develop and share knowledge for the benefit of research on municipal health and care services, and eventually, the quality of municipal health and care in Europe. Main activities of the project will be network development, capacity building, providing a platform where multiprofessional primary care research activities can take place and publishing position papers.

## The PRIMORE Project

The Norwegian Council for Research (NCR) has recently funded a research network for three years starting 1 October 2018, led by the Centre for Care Research (CCR), Norway, with the European Forum for Primary Care (EFPC) and the Research Unit for General Practice at University of Bergen, Norway, as partners. The objective of the new network, PRImary care MultiprOfessional REsearcher network (PRIMORE), is to create opportunities for primary care researchers and long-term care researchers from different disciplines, sectors and countries to develop and share knowledge for the benefit of research on municipal health and care services, and eventually, the quality of municipal health and care in Europe. Although both long-term care research and primary care research are multiprofessional by nature and researchers within both traditions experience several of the same challenges as obstacles for good and well-integrated services for local communities, they mostly attend separate conferences, publish in separate journals and, to some extent, employ different methodological designs.

## Why multiprofessional collaboration is needed

Multiprofessional collaboration is particularly important for the management of long-term conditions, often with multimorbidity, and for conditions that involve multiple health problems, but also prevention and health promotion, at the crossroads of health care and social care (Samuelson, *et al.*, 2012).

Insufficient coordination is a major cause for lack of responsiveness and poor efficiency of health systems. Patients themselves or their social system have to coordinate care in the absence of good collaboration between professionals (Samuelson, *et al.*, 2012). A strategy of community-oriented health and care research, where researchers from different traditions join forces and learn from each other, aiming at integration of care and the enhancement of coordination among primary care and long-term care services, is important. This strategy can have beneficial consequences for development of the services researched, the community-oriented services, and further facilitate coordination between the health and care services at the municipal level and other levels and types of services (Thomas, *et al.*, 2015).

There is a strong relationship between health care systems with a strong primary care on one side and better health and lower costs on the other (Schäfer *et al.*, 2015; Starfield, Shi & Macinko, 2005; WHO, 2015a, 2015b). Countries with a strong primary care system and established multiprofessional collaboration in primary care teams tend to develop more comprehensive models to manage complex care problems, ensure access to services, continuity of care, coordination and integration of services (Samuelson, *et al.*, 2012). Chronic conditions and multimorbidity can be treated more effectively by different closely collaborating health care workers among whom tasks may be reshuffled (Armitage, *et al.*, 2009; De Maeseneer & Boeckxstaens, 2012). And countries that have a better professional infrastructure or a stronger academic tradition in primary care are more often ahead of others in developing interprofessional collaboration (Kringos, *et al.*, 2015). There is, however, a need for comprehensive studies at the macro (system integration), intermediate (professional and organizational integration) and micro levels (clinical integration) to better understand the relationship between design and provision of primary care and health outcomes (Tallia *et al.*, 2003; Valentijn, *et al.*, 2013). At the micro level, an integrated interprofessional approach by a co-operating team is essential to address the complexity of multiproblem situations. These interprofessional teams are organized as multidisciplinary practices (e.g. Community Health Centres), or as networks of different disciplines (Szreter & Woolcock, 2003; De Maeseneer, 2017). At the intermediate level (the community), the primary care sector is accountable for health of a defined population. At the macro level, strong primary health care systems are associated with a more equitable distribution of health in the population (De Maeseneer, 2017). Each position paper will deal with service integration at all these three levels.

The EFPC position paper on interprofessional collaboration defined major areas in need of improvement as professional education, occupational structure of health care and issues of skill mix at different levels (Samuelson, *et al.*, 2012). In concordance with this definition, PRIMORE aims to contribute to the body of scientific knowledge by strengthening research capacity in different European states, offering a forum for exchange, facilitating capacity building initiatives and bringing together fragmented research initiatives, stressing systems and service research. Moreover, important scientific benefits can be expected, such as a growing evidence base for community-oriented health and care services, including a measure for community orientation, resulting in applications of research-based models that will help decision makers to reform (health) care systems more effectively on the basis of proven models. Furthermore, the European capacity for community-oriented health and care services research is expected to grow overall. Community-oriented primary care integrates individual and population-based care, blending clinical skills of practitioners with epidemiology, preventive medicine and health promotion, minimizing the separation between public health and individual health care (Rhyne *et al.*, 1998). For this approach to be successful, multiprofessional teams, including community members, are essential.

The network will contribute to the response of community-oriented services in Europe to the challenges facing health care systems, such as epidemiological transition, the aging population and increased migration. The response of primary care to the changing needs of the populations should be based on solidarity, respect for diversity and building bridges instead of walls with a special emphasis on ‘connectedness’ (De Maeseneer, 2017). The network’s research will hence also relate to these basic societal values and sustainability. It will contribute to the knowledge base related to improving cost-effectiveness through better organized primary care systems and greater responsiveness to health needs of populations through community orientation and user involvement.

## Objective and activities

The main objective of this proposed project, to develop an international, multiprofessional network in community-oriented health and care research, will be pursued through *five main activities:*

1. Develop models for a multiprofessional health system and services research in primary health care that connects research, policy and practice
2. Support and initiate exchange of knowledge and experience
3. Support of research in countries with low capacity
4. Undertake dissemination activities aimed at researchers, policy makers and service providers
5. Develop forums for cooperation between researcher, policy makers and practice.

These activities are specified in the following deliverables.

### Network development

The work of network development will be led by the CCR, Norway, in close cooperation with EFPC, of which CCR is an institutional member and the Research Unit for General Practice at University of Bergen, Norway. The EFPC has several major research institutes focused on community health (including those with WHO Collaborating Centre accreditation) as its members and has close links with the research networks of the different professional groups like EGPRN (European General Practice Research Network), ENOTHE (European Network of Occupational Therapy in Higher Education), PGEU (Pharmaceutical Group of the European Union) and EFAD (European Federation of the Associations of Dieticians) of which the PRIMORE network will benefit from. All partners have extensive research connections on a global level, which provides plenty of opportunities to link up with similar networks in Australia, Canada, USA, South Africa, etc. The EFPC is partner in several European initiatives, such as the European Innovation Partnership on Active and Healthy Ageing, Health Care Professional Working Party of the European Medicine Agency and EU Health Policy Platform, and it secures mutual information channels with these initiatives.

### Information and knowledge exchange including the establishment of a multiprofessional primary care research infrastructure

Regular meetings will be organized for members of the network. A password-protected online platform, including the creation of new scenarios of knowledge transfer, will be established. On-site and online meetings will deal with special themes, presentations by members, discussion of important research topics and joint initiatives. The online platform (PIE) will provide institutional support for senior members and their institutions to start health services research initiatives. PIE (Problems, Ideas & Experiences) facilitates the exchange of knowledge and capabilities. It will break through organizational walls, collect ideas which respond to the current challenges of community health, foster quick dissemination and implementation of new solutions and improvements and discover hidden talents, creating a community of innovators motivated to work creatively (Jakab, 2018). This online platform will facilitate PRIMORE to connect other research networks mentioned above.

In addition, the network will support the development of local primary care networks in participating countries, which will aid the dissemination of research results at the local level and help feed back the results into the research community. Dissemination includes the production of position papers, scientific publications, organization of international study visits, messages through social media and organization of conferences.

### Capacity building

The network aims to organize master-classes for early career researchers to further develop their professional and research skills. Senior participants and their institutes will provide major inputs to these activities. A special focus will be on states in the Central and Eastern Europe (CEE), where the EFPC has a solid network among research institutes. The EFPC has strong connections to European scientific journals concerned with quality in primary care and primary care research. This will provide young researchers and researchers from CEE countries an academic platform for publication.

### Scientific programme and innovation

The scientific programme of the network will firstly be multiprofessional, bridging the mono-professional approaches prevailing in primary care research and long-term care research. Inputs will be secured from different professionals (general practitioners, nurses, physiotherapists, odontologists, psychologists, occupational therapists, pharmacists, social work, midwifery, etc.) active in primary care, brought together in research designs by a range of academics from different origin (health care professionals, anthropologists, sociologists, demographers, epidemiologists, etc.). Primary care will be studied in its context, and interfaces to secondary care, social services and patients/citizens will be taken into account. There are important representatives of patient/citizen networks among the EFPC members.

Secondly, it will promote research in areas of primary care that have been poorly studied until now. The conceptual framework of interdisciplinary collaboration in the context of health care delivery as described by EFPC 2012 position paper provides a useful baseline for developing such multiprofessional research projects in primary care (Samuelson, *et al.*, 2012). Thirdly, it will be cross-sectoral, for example by working together with researchers of working life on the issue of recruiting and retaining a competent workforce and good employment and integration of an increasingly multicultural workforce within primary care services.

Professional exchanges in this network will focus on study designs, which take into account the typical features of primary care such as an integrated provision through teamwork, a generalist approach to a wide range of undifferentiated health problems and coordination of care even after referral from hospital. Position papers, aiming to facilitate the international exchange between researchers, practitioners and policy makers, published on several sub-domains, play an important role in this respect.

Three specific topics which will be analysed are (A) ‘Healthy Aging, new concepts at community level’, (B) ‘Mental Health’ and (C) ‘Organization of Primary Care including practice size, interprofessional collaboration and integration of social and health care’. Multilevel work-groups led by CCR and other member institutes of the EFPC with a strong track record will be initiated. These groups will meet each other through the online platform and work on the production of three position papers related to the above mentioned topics (A–C) during the three years of network support. A version of each position paper will be published in a scientific journal, taking advantage of an agreement between the EFPC and the scientific journal of *Primary Health Care Research & Development* (PHCR&D).

Work groups will be established related to different themes to stimulate local and national initiatives. These work groups consist of both researchers and representatives of professional associations and client platforms and will be facilitated via the EFPC website with the specific webpage for the network.

Finally, the network will organize one PhD summer course on ‘Community-Oriented Primary Care Services’. This summer course aims to contribute to the research capacity concerning primary care in Norway and the rest of Europe, attracting young health service researchers to the field of primary care research.

While the CCR is formally leading the network project and EFPC has a particular responsibility for the web platform and other relevant infrastructure for network meetings, all three partners are responsible for the academic output of the project like the position papers, the summer school and other dissemination and education activities.

## Conclusion

The PRIMORE research network project aims at bringing together researchers with different backgrounds and from a wide range of professional groups within the fields of primary care research and long-term care research to develop and share knowledge for the benefit of research on municipal health and care services, and eventually, the quality of municipal health and care in Europe. As part of this venture, education of students and young researchers and development of position papers will take place.
